# Epidemiology, Diagnosis, and Treatment of HIV-Associated Non-Hodgkin Lymphoma in Resource-Limited Settings

**DOI:** 10.1155/2012/932658

**Published:** 2012-03-26

**Authors:** Matthew Ulrickson, Oliver W. Press, Corey Casper

**Affiliations:** ^1^Department of Medicine, University of Washington/Fred Hutchinson Cancer Research Center, Seattle, WA 98109, USA; ^2^Clinical Research Division, Fred Hutchinson Cancer Research Center, Seattle, WA 98109, USA; ^3^Vaccine and Infectious Disease, Public Health Sciences, and Clinical Research Divisions, Fred Hutchinson Cancer Research Center, Seattle, WA 98109, USA

## Abstract

Lymphoma was a common complication of HIV infection in the pre-antiretroviral era, and the incidence of HIV-associated lymphoma has dropped dramatically since the introduction of combination antiretroviral therapy (cART) in resource-rich regions. Conversely, lymphoma is an increasingly common complication of HIV infection in resource-limited settings where the prevalence of HIV infection is high. Relatively little is known, however, about the true incidence and optimal treatment regimens for HIV-associated lymphoma in resource-poor regions. We review the epidemiology, diagnosis, and treatment of HIV-associated non-Hodgkin lymphoma in developing nations and highlight areas for further research that may benefit care in both settings. Examples include risk modification and dose modification of chemotherapy based on HIV risk factors, improving our understanding of the current burden of disease through national cancer registries, and developing cost-effective hematopathological diagnostic strategies to optimize care delivery and maximize use of available chemotherapy.

## 1. Introduction

An association between the acquired immunodeficiency syndrome (AIDS) and lymphoma was first suggested in 1982 after four young men in San Francisco with severe immunodeficiency were diagnosed with a “Burkitt-like lymphoma” [1, 2]. Since that time, HIV has been identified as the causative agent of the underlying immunodeficiency and non-Hodgkin lymphoma (NHL) was designated as an AIDS-defining malignancy [3]. While most of the early descriptions of the emerging immunodeficiency syndrome were reported in patients living in the United States, most of the burden of HIV disease now affects resource-limited nations, with approximately two-thirds of HIV-positive individuals living in sub-Saharan Africa and only 8% within Western nations [4]. The discovery and widespread use of combination antiretroviral therapy (cART) in resource-rich countries has both decreased the incidence of HIV-associated lymphoma and improved its prognosis [5, 6]. While the availability of cART has improved in resource-poor nations due to extraordinary recent efforts, similar changes in the incidence and outcome of HIV-associated lymphomas have not yet been noted. Therefore, increased efforts should be dedicated to improving the diagnosis, supportive care, and treatment of this disorder in these nations.

## 2. History and Epidemiology

The incidence of the three AIDS-defining NHLs, diffuse large B-cell lymphoma (DLBCL), primary CNS lymphoma, and Burkitt lymphoma (BL), increased steadily in the United States between 1981 and 1990, at which time the incidence rate leveled off and began declining with the widespread availability of cART in 1996 [7]. Over 25,000 Americans with HIV have been diagnosed with NHL since the beginning of the HIV pandemic [8].

**Figure 1:**
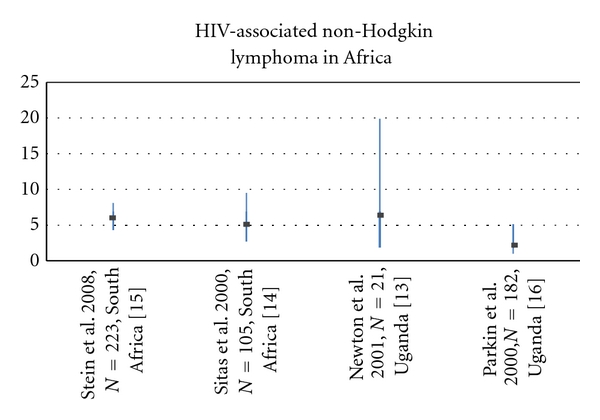
Odds Ratios (ORs) with 95% Confidence Intervals (CIs) for the association between NHL and HIV infection in sub-Saharan Africa.

In Africa, the AIDS epidemic was first reported by Clumeck et al. and Van De Perre et al. in 1984 when they described a group of Africans cared for in Belgium with profound immunosuppression and a series of patients in Kigali, Rwanda with infectious complications or generalized Kaposi sarcoma, respectively [9, 10]. Nearly equal numbers of men and women were reported in these articles, in contrast to the predominately male epidemiology described early in the United States HIV experience. The incidence of NHL in sub-Saharan Africa did not increase as markedly early in the HIV epidemic when compared to the increase seen in the US HIV population. This has been attributed to the higher rate of infectious complications that were seen in HIV-infected patients in resource-limited nations, which prevented the subsequent development of malignancies [11]. This decreased incidence may also simply reflect underreporting, attributable to more limited pathology services in combination with differences in the epidemiology of adenopathy in sub-Saharan Africa. Since the most common empiric clinical diagnosis of persistent lymphadenopathy is tuberculosis and not lymphoma, many cases may have gone unnoticed without biopsy confirmation [12]. By the mid-1990s, an increase in lymphoma risk associated with HIV was noted and at present most studies report a 5-6-fold increased risk for development of NHL in HIV-infected individuals living in Africa as seen in Figure 1 [13–16].

**Figure 2:**
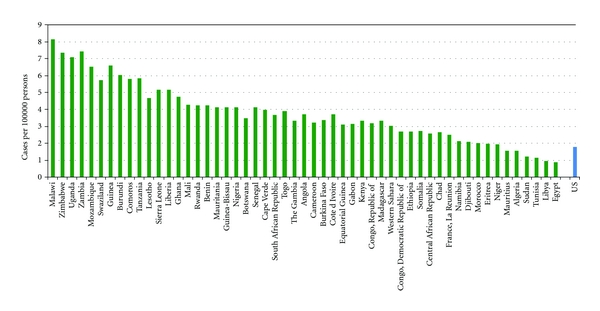
Incidence of non-Hodgkin lymphoma in Africa compared to the United States. Data courtesy of GLOBOCAN 2008.

On average, patients in Africa present with HIV-associated complications at a higher CD4 count compared to those in resource-rich nations. With current supportive care, the burden of HIV-associated NHL is currently estimated at about 15,000 per year in the equatorial belt of Africa alone [17, 18]. The true incidence is believed to be higher, due to continued limitations in diagnosis and presentation to medical care, though the HIV-associated risk for NHL still does not seem to be as high as in resource-rich nations in the pre-cART era. Data from the International Agency for Research on Cancer shows that the incidence of NHL in most countries of Africa exceeds that of the USA by 2–5-fold. Although cancer registry data in Africa do not routinely capture the HIV status of new cancer cases, Figure 2 clearly illustrates that the incidence of NHL is highest in African countries with a high prevalence of HIV infection, though part of this trend may reflect improved diagnosis associated with HIV clinics. 

The Kampala Cancer Registry is one of the oldest continually operating cancer registries in Africa and one of two which are WHO-certified. This registry was created in 1954 and enables an estimation of the impact of HIV infection on rates of malignancy in the area and serves as a tool for ongoing research [19]. The incidence of NHL in Kampala has increased 6.7% annually in men and 11% annually in women since the beginning of the HIV pandemic [20]. Linking HIV and cancer registries in Uganda showed that the incidence of NHL was 6.7-fold higher among HIV-infected persons in Uganda compared with those who are HIV negative, lending credence to the hypothesis that the increase in NHL incidence in sub-Saharan Africa is in part fueled by the HIV pandemic. 

Comprehensive data on survival after a diagnosis of NHL in HIV-positive persons are lacking in both resource-rich and resource-poor regions. However, two recent studies highlight the discrepancies in survival between patients in these two settings. In the USA, 47% of HIV-positive persons diagnosed with non-CNS NHL were alive 2 years after their diagnosis [21], which was nearly identical to the survival among HIV-positive Ugandans who received cART [22]. Of note, however, is the fact that 100% of HIV-positive Ugandans with NHL who failed to receive cART died within one year of NHL diagnosis, highlighting the importance of the comanagement of HIV and cancer.

Increasing the number of such registries and the quality of data collected will contribute significantly to improving our understanding of the epidemiology of lymphoma in Africa. Collecting information on HIV-associated malignancies at clinics that distribute antiretrovirals is one potential way to increase the available data.

## 3. Diagnosis

A necessary complement to expanded cancer registry data in resource-poor nations is continued improvement in diagnostic accuracy, especially of hematological neoplasms. The accurate diagnosis of lymphomas can be challenging, even for pathologists in developed nations without hematopathological specialization. Diagnostic modifications were made in 20% of a series of submitted lymphoma cases in the United Kingdom after central review. After evaluation of associated clinical data in each of the cases, the authors concluded that clinical management likely would have differed in about half of the cases with diagnostic discrepancies [23]. Similarly, the diagnosis of lymphoma in developing nations is challenging and significant modifications in final diagnosis have been reported after hematopathology review. A retrospective analysis of 207 NHL cases in Kenya resulted in the histologic reclassification of 41% of the cases [24]. An additional study by Parkins and authors reviewed 150 cases of suspected lymphoproliferative disorders from two teaching hospitals in Ghana at the time of diagnosis. After this review, modifications were made to the diagnoses of 44% of patients, and alterations in the subsequent clinical management in 31% of the 150 patients as a result. Some of the final diagnoses in this group included nasopharyngeal carcinoma and tuberculosis, with marked differences in management resulting [25]. The rates of significant diagnostic changes do vary between studies, however. In a prospective trial of combination chemotherapy in HIV-associated lymphoma in East Africa, pathological review led to changes in diagnosis in only 6% of the 32 patients with tissue samples available. Additionally, when the outcomes of patients with pathological confirmation were compared to those that did not undergo pathological review, no differences in outcome were noted [26].

As optimal therapies become more varied for each lymphoma subtype, it is likely that the clinical significance of diagnostic accuracy will increase. An initial attempt to improve this diagnostic accuracy in a cost-effective manner was recently reported by Naresh and colleagues through a description of a diagnostic algorithm for Burkitt lymphoma. They described a three-tiered method for diagnosis that involved increasing numbers of immunohistochemical tests in each tier if the diagnosis remained unclear. They were able to confirm a diagnosis of BL versus DLBCL in 82% of the cases using the first phase of studies (CD10, bcl2, and morphology) and in 92% of cases with the addition of testing for Ki-67, CD38, and CD44 [27]. This type of data can be used to guide developing health systems in prioritizing the diagnostic procedures that are most cost-effective.

The benefit of immunohistochemistry, not routinely available in resource-limited regions at present, extends beyond its assistance in confirming a diagnosis in this age of increasing numbers of targeted therapies. Discovering immunophenotypic differences of lymphomas in varying patient populations may promote discovery of novel targeted agents as well as further evaluation of current agents in clinical trials, even at the present time. For example, Tumwine and authors described the immunohistochemical features of 119 cases of lymphoma in Kampala, Uganda. 37% of the Burkitt lymphoma cases were CD30 positive [28], twice the rate of 18% noted in a similar series from the United Kingdom [29]. With the recent development of an antibody-chemotherapy conjugate that targets CD30, such a finding could impact treatment outcomes in this population [30].

## 4. Chemotherapy in the Pre-cART Era

At the present time, standard therapy for HIV-associated lymphoma in Africa does not include targeted therapy, such as rituximab, as current medication costs are prohibitive. Therefore, treatment is delivered with standard cytotoxic agents alone. Because of the similarities between the current patient population in resource-limited nations and patients in resource-rich nations in the pre-cART era, a historical review of chemotherapy for HIV-associated lymphomas is helpful for identifying potential areas of further study.

Initially, the treatment of AIDS-associated lymphoma in the United States achieved complete response (CR) rates of 53% with combination chemotherapy usually involving CHOP (cyclophosphamide, doxorubicin, vincristine, and prednisone), though these responses were tempered by a rate of relapse of 54%. Additionally, significant infectious complications were noted in 42% of the cohort, which contributed to a mortality rate of 85% during a three-year study by Ziegler and colleagues reported in 1984 [31].

The next generation of chemotherapy trials in AIDS-related lymphoma studied more aggressive chemotherapy combinations in recognition of the fact that such cases typically presented at a more advanced stage, had a higher grade at presentation, an increased risk of relapse, and an increased frequency of extranodal disease compared with non-AIDS-related lymphomas. For example, Gill and authors compared M-BACOD (high-dose methotrexate, bleomycin, doxorubicin, cyclophosphamide, vincristine, and dexamethasone) with an even more intensive regimen using high-dose cytarabine and high-dose methotrexate in combination with other agents. Rates of complete remission were 54% and 33% in the two groups, respectively, and the overall survival was 11 months in the M-BACOD group compared to 6 months in the cytarabine/methotrexate arm. This trend towards shorter survival was attributed to a higher rate of opportunistic infections in the cytarabine/methotrexate arm, with more than 75% developing this complication [32]. Similarly, Kaplan and colleagues found a high-dose regimen, COMET-A (cyclophosphamide, vincristine, methotrexate, etoposide, and high-dose cytarabine), was associated with decreased overall survival when compared to standard therapy such as CHOP in patients with HIV-associated lymphoma. Outcomes in both of these studies were best predicted by pretreatment CD4 count, performance status and presence of extranodal disease [33]. Since infectious complications overwhelmed any potential advantage to these aggressive regimens, less-intensive chemotherapy regimens were studied next.

A 1997 study by the AIDS Clinical Trials Group (ACTG 142) therefore compared lower-dose m-BACOD to standard dose m-BACOD with GM-CSF support. The lower-dose regimen was associated with decreased hematological toxicity and similar rates of response. CR rate in the low-dose arm was 39%, compared to 52% in the standard-dose arm while median overall survival was 35 weeks compared to 31 weeks, respectively. Neither of these differences was statistically significant [34]. The survival impact of regimen-related toxicity in the treatment of HIV-associated lymphomas in the pre-cART era was further clarified by a trial conducted by Mounier and colleagues where the selection of chemotherapy was adjusted based on clinical risk factors present at diagnosis. Subjects treated between 1993 and 1999 were stratified based on ECOG performance status of 2–4, a prior clinical diagnosis of AIDS, and a CD4 count less than 100 cells/*μ*L. Patients with none of these risk factors received one of two aggressive chemotherapy regimens and no differences in overall survival were noted between 4 cycles of CHOP (cyclophosphamide, doxorubicin, vincristine, and prednisone) and 3 cycles of ACVBP (doxorubicin, cyclophosphamide, vindesine, bleomycin, and prednisolone). Patients with one risk factor received either standard-dose or low-dose CHOP, again without any difference in overall survival. The third group, with either 2 or 3 risk factors, received either 4 cycles of low-dose CHOP or 12 cycles of vincristine and prednisolone. There were no differences in overall survival between regimens in any of the risk groups, though lymphoma-specific outcomes were improved in the more aggressive arms of each subset. It should be noted that after 1996 most patients were started on cART, and this subset composed about one-third of the entire study cohort. While cART did decrease the number of patients with 2-3 risk factors and improve overall survival, it did not lead to any significant differences between chemotherapy regimens in risk-stratified patients [35]. Therefore, a decrease in regimen intensity and dose adjustment by hematological parameters for high-risk patients should be considered when treating lymphoma in patients not on cART. 

The balance of minimizing myelotoxicity and infectious risk while optimizing lymphoma therapy has an even narrower therapeutic window in resource-limited nations due to the decreased availability of supportive care measures such as broad-spectrum antibiotics and the high rates of baseline cytopenias. Concurrent use of other supportive medications may be a major contributor to the high rates of cytopenias in African patients with HIV infection. For example, in a study of 498 patients started on antiretroviral therapy in Cote d'Ivoire, the observed 24% rate of grade 3-4 neutropenia was ascribed to the combined administration of trimethoprim/sulfamethoxazole and zidovudine (AZT). While the neutropenia usually resolved with discontinuation of the TMP/SMX, the omission of this prophylactic therapy increased the risk of infectious complications, a risk that would likely increase further in the setting of cytotoxic chemotherapy [36]. This infectious risk was demonstrated clinically in an early trial of chemotherapy for HIV-associated Burkitt lymphoma in Africa. This cohort of BL patients older than 16 years old, treated between 1993 and 1996, experienced a treatment-related mortality rate of 46% with a median overall survival of 15 weeks [37].

## 5. Chemotherapy in the cART Era

After the discovery and widespread use of cART in resource-rich countries, overall survival in HIV-associated lymphoma improved, in one study from an average of 6 months to 20 months [6], and more aggressive treatment regimens were once again considered. With the additional immunological support enabled with cART, the next major trial of chemotherapy in HIV-associated lymphoma compared CHOP to rituximab-CHOP combination therapy and was reported by Kaplan and authors as part of the AIDS Malignancy Consortium trial 010. This study found a trend towards increased efficacy (CR rate of 58% versus 47%) in the rituximab plus chemotherapy arm, however, also noted a significantly increased risk of treatment-related death in the combination arm of 14% compared to 2%. This increased risk of death was attributed to increased infectious complications during the treatment course and, on additional analysis, seemed to predominate in patients with CD4 counts <50 cells/*μ*L. This finding supports the importance of risk stratification by CD4 count, even in patients on cART at the start of chemotherapy [38].

For a time, this report led to decreased use of rituximab therapy in patients with HIV-associated lymphoma. However, additional data was recently provided by Dunleavy and colleagues [39] and Sparano and colleagues [40] in a pair of articles published in 2010. In these studies, rituximab therapy was used to treat HIV-positive patients with DLBCL and NHL, respectively. There were no treatment-associated infectious deaths noted in the study by Dunleavy, supporting the safety of rituximab in this setting. Additionally, this study demonstrated other methods aimed at decreasing the duration of associated immunosuppression and myelotoxicity with chemotherapy regimens. The authors utilized a decreased number of cycles of infusional EPOCH in combination with “dose-dense” rituximab (given on days 1 and 5). The EPOCH was dose adjusted based on hematological parameters per protocol prior to each cycle of therapy, thereby regulating the average neutrophil nadir. Second, the study utilized interim FDG-PET scans to guide the number of chemotherapy cycles, with one cycle given after the first negative PET-CT. By using this strategy, about 80% of the patients received only three cycles of therapy with the highest number of received cycles being 5, delivered in 12% of the subjects. The CR rate was 91% with 5-year PFS and OS of 84% and 68%, respectively. Of the 10 deaths on study, half were due to lymphoma and 5 occurred while subjects remained in remission. 3 of these deaths were due to opportunistic infection and 1 developed a secondary Burkitt lymphoma. The immediate application of this treatment regimen in resource-limited settings is prevented for multiple reasons. First, a lack of hospital beds and hospital staff relative to the large numbers of patients in need of treatment prevents delivery of five days of infusional EPOCH. Second, while this regimen is dose adjusted for hematological parameters, it is delivered with G-CSF support, a therapy that is not available in most resource-poor settings. Third, PET scans, and even CT scans, are not available in routine clinical practice at the present time to rationally select patients that may safely receive fewer cycles of chemotherapy.

Even with these limitations, however, several principles can be incorporated to improve care in resource-poor settings. Dose adjustment has been investigated in trials of chemotherapy in sub-Saharan Africa with some success. One such study, and the first prospective trial investigating chemotherapy for HIV-associated lymphomas in Africa, was performed by Mwanda and colleagues, reported in 2009 [26]. This study utilized a dose-modified oral chemotherapy regimen based on a United States trial that was completed before the availability of antiretroviral therapy [41]. The dose-modified regimen included lomustine (50 mg/m^2^ day 1, cycle 1 only), etoposide (100 mg/m^2^ days 1–3), cyclophosphamide (100 mg/m^2^ days 22–26), and procarbazine (100 mg/m^2^ days 22–26). As the initial regimen required G-CSF support, doses were decreased in the protocol to prevent this requirement. Additional modifications during therapy were also outlined, in a manner similar to the infusional EPOCH regimen. These adjustments led to a 50% dose reduction in all medications for an absolute WBC count less than 3,000 cells/*μ*L or a platelet count under 100,000 cells/*μ*L. For WBC count less than 1,500 cells/*μ*L or platelets under 50,000 cells/*μ*L doses were held until counts improved. If counts did not improve within three weeks, subjects were removed from the protocol—though this did not occur in any subjects on protocol. Median CD4 count in the 49-patient cohort was 198 cells/*μ*L, 37% of patients were on antiretroviral therapy, and most had a history of previous opportunistic infection. 65% of the patients completed the two cycles determined by the protocol and a 6% treatment-related mortality was noted during the study, a significant improvement from previous studies. The reported CR rate was 58% with 78% having an objective response, numbers that are comparable to pre-cART studies in resource-rich nations. Median overall survival was 12 months, though a large variance was noted in patients on cART compared to no retroviral therapy with a median overall survival in the latter group of about 6 months. 

This study serves as an example that prospective chemotherapy trials can be completed in resource-limited settings. It also identifies additional benefits from such studies, which may aid the management of HIV-associated lymphoma in resource-rich nations. First, this study by Mwanda and colleagues in East Africa is notable for its inclusion of female participants. In contrast to the >85% male composition of many of the other major trials in the field [6, 35, 39, 40] this cohort was composed of 60% women. Second, the chemotherapy regimen was composed of some medications that are known to cross the blood-brain barrier (lomustine, procarbazine) and without additional intrathecal therapy the rate of CNS relapse (6%) was similar compared to other trials performed prior to widespread availability of cART. Further investigation of the use of CNS-active agents in HIV-associated lymphoma therapy may be warranted, especially in situations where delivery of intrathecal therapy is difficult.

## 6. Supportive Care

Since platelet transfusions are currently not routinely available in most resource-limited nations [42], conditions exist to study chemotherapy regimens that decrease the need for transfusion and to evaluate adjunctive therapies that may decrease bleeding complications in the setting of thrombocytopenia such as aminocaproic acid [43]. This could improve our understanding of the optimal management of patients in resource-rich nations that have an objection to receiving blood products.

Another aspect of the regimen used by Mwanda that may warrant further study is the avoidance of corticosteroids, even though they are known to be active lympholytics. The rationale for not including corticosteroids in the study regimen is avoidance of potential exacerbation of concurrent Kaposi sarcoma (KS) and HHV-8 infection, an important consideration as the prevalence of KS is highest in East Africa [44]. Similarly, increased immunosuppression due to corticosteroid administration could be associated with increased toxicity in patients with HIV due to other infectious comorbidities. The increased risk of reactivation or infection with *M. tuberculosis* is a consideration when balancing the risks and benefits of corticosteroids in high prevalence regions [45]. Hepatitis B virus (HBV) is another infection that has been shown to reactivate more commonly when corticosteroids are included in chemotherapy regimens for lymphoma [46] and is well known to reactivate during rituximab therapy if viral prophylaxis is not administered in previously exposed patients [47]. There is a 13% rate of detectable serum hepatitis B DNA in patients admitted to Mulago Hospital in Uganda, [48] which could enable further clarification of the magnitude of risk associated with HBV reactivation compared with the treatment benefit of corticosteroids. These results could improve clinical decision-making concerning the ideal chemotherapy regimen to use in the setting of concurrent HIV and HBV infection.

## 7. Conclusions

While a large portion of the improvements in treatment of HIV-associated lymphomas in resource-limited nations will occur as a result of earlier diagnosis, increased access to cART, and optimal treatment of concurrent infectious disease, significant opportunities to improve the hematological management of such cases also exist. Some of these advances may also benefit patients with HIV-associated lymphoma in resource-rich nations. Examples include improved risk modification and dose modification of chemotherapy, improved diagnostic capabilities with eventual implementation of targeted therapies based on the immunophenotypic profile, and an increased understanding of optimal supportive care for patients with infectious comorbidities.

The cost of cancer care is often raised as a barrier to increasing access in resource-limited settings. On the contrary, however, it is the cost of inaction that is estimated to be much more significant. The Global Task Force on Expanded Access to Cancer Care and Control estimated that the world could have saved $131 billion in 2010 by investing in cancer treatment and prevention to limit the associated disability-adjusted life years [49]. Similarly, the 2011 World Economic Forum report identified noncommunicable diseases as a global economic threat, framing health care as an investment instead of simply expenditure [50]. This perspective, in combination with the fact that “cancer now kills more people each year in (low and middle-income countries) than AIDS, tuberculosis, and malaria combined” [51] highlights the ethical and financial imperative to improve the care of lymphoma globally and serves as a call to action for the hematology community.
